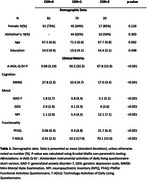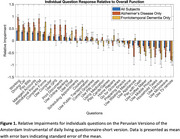# Divergent Functional Capacity in Alzheimer's Disease and Frontotemporal Dementia: Insights from the Peruvian Adaptation of the Amsterdam Instrumental Activities of Daily Living Questionnaire

**DOI:** 10.1002/alz70857_104952

**Published:** 2025-12-25

**Authors:** Gregory Brown, Diego Bustamante‐Paytan, Maria Fe Albujar‐Pereira, José Carlos Huilca, Katherine Aguero, Graciet Verastegui, Zadith Yauri, Pamela Bartolo, Daniela Bendezu, Karol Melissa Lipa‐Pari, Rosa Montesinos, Nilton Custodio

**Affiliations:** ^1^ Instituto Peruano de Neurociencias, Lima, Lima, Peru; ^2^ University of California, San Francisco, San Francisco, CA, USA; ^3^ Equilibria, Lima, Lima, Peru; ^4^ Instituto Peruano de Neurociencias, Lima, Peru; ^5^ Hospital Nacional Cayetano Heredia, Lima, Lima, Peru; ^6^ Universidad de San Martín de Porres, Facultad de Medicina, Centro de Investigación del Envejecimiento, Lima, Lima, Peru; ^7^ Unidad de Investigación y Docencia, Equilibria, Lima, Peru., Lima, Lima, Peru

## Abstract

**Background:**

Instrumental activities of daily living (IADL) involve activities with higher cognitive demands, such as meal preparation, shopping, home maintenance, and financial management. IADL disturbances often emerge in early disease stages, affected by cognitive deficits regardless of dementia diagnosis. There are distinct functional profiles across Alzheimer's disease (AD) and frontotemporal dementia (FTD) syndrome. This study explores everyday functioning between FTD and AD dementia, focusing on individual daily activities using the Peruvian adaptation of Amsterdam IADL Questionnaire short version (A‐IADL‐Q‐SV‐P).

**Methods:**

Participants were recruited at the Instituto Peruano de Neurociencias‐IPN in Lima, Peru between June 2020 and December 2023. Eligible patients with AD and FTD were invited to participate, according to McKhann criteria for AD and Rascovsky et al. criteria for FTD. Dementia severity was assessed using the Clinical Dementia Rating scale (CDR), including CDR 1 and 2. We use the Spanish version of A‐IADL‐Q‐SV which can be obtained for free by registering with the developers of the instrument (https://www.alzheimercentrum.nl/professionals/amsterdam‐iadl/). We then identified which activities had worse impairments relative to a person’ total score and compared these relative impairments between AD and FTD using independent samples t‐tests.

**Results:**

Of 171 recruited individuals, 81 were cognitively normal, 54 had Alzheimer's disease and 36 had frontotemporal dementia. Work, driving and paying bills were the most impaired IADLs across both syndromes, while using kitchen appliances and remote controls were least impaired. People with FTD showed worse impairments in cooking (*p* <0.001) and using appliances (*p* = 0.017) compared to AD patients. ATM cash withdrawals (*p* = 0.027) were more impaired in AD dementia compared to FTD.

**Conclusion:**

Findings revealed that FTD patients were significantly more impaired on tasks such as cooking and using appliances compared to AD patients, likely due to early executive dysfunction. These results will be important for carers and clinicians developing effective strategies to manage functional deficits.